# Patch Based Multiple Instance Learning Algorithm for Object Tracking

**DOI:** 10.1155/2017/2426475

**Published:** 2017-02-22

**Authors:** Zhenjie Wang, Lijia Wang, Hua Zhang

**Affiliations:** ^1^Department of Information Engineering and Automation, Hebei College of Industry and Technology, Shijiazhuang, China; ^2^Faculty of Electrical & Electronics Engineering, Shijiazhuang University of Applied Technology, Shijiazhuang, China

## Abstract

To deal with the problems of illumination changes or pose variations and serious partial occlusion, patch based multiple instance learning (P-MIL) algorithm is proposed. The algorithm divides an object into many blocks. Then, the online MIL algorithm is applied on each block for obtaining strong classifier. The algorithm takes account of both the average classification score and classification scores of all the blocks for detecting the object. In particular, compared with the whole object based MIL algorithm, the P-MIL algorithm detects the object according to the unoccluded patches when partial occlusion occurs. After detecting the object, the learning rates for updating weak classifiers' parameters are adaptively tuned. The classifier updating strategy avoids overupdating and underupdating the parameters. Finally, the proposed method is compared with other state-of-the-art algorithms on several classical videos. The experiment results illustrate that the proposed method performs well especially in case of illumination changes or pose variations and partial occlusion. Moreover, the algorithm realizes real-time object tracking.

## 1. Introduce

Object tracking is an important topic in computer vision, and it has attracted more and more attention [1]. Recently, researchers have studied the issue and proposed many excellent algorithms. However, the object tracking is still a challenging task because there are noise, pose variations, illumination changes, occlusion, and so on [2].

An efficient appearance model plays an important role in object tracking system. Studies categorize the appearance models into two classes: generative appearance model and discriminative appearance model [3]. Generative appearance algorithms [4–7] take the tracking problem as searching for the region with the maximum score. The methods represent an object in a particular feature space such as edge, color, and HOG. By using the appearance model, the algorithms search for interesting regions with the minimal error in the successive frames. It is demonstrated that the algorithms have achieved favorite tracking results. However, the generative algorithms take into account only the feature of object but ignore the background information which are helpful for recognizing object from background [1].

The discriminative methods [8–12] formulate the tracking problem as learning a discriminative classifier which is updated over time to separate object from background. The classifier updates its parameters considering the information from both the object and background to handle the issues of appearance changes as tracking evolves. Grabner et al. [13] presented an online boosting feature selection algorithm for object tracking. However, only one positive sample (the tracking result) is used for classifier updating. Once the tracking result drifts away from the ground truth, the positive sample is inaccurately cropped [14]. Therefore, the interference from background will be introduced into the classifier, which leads to tracking failure. To handle this problem, Grabner et al. [15] presented a semisupervised object tracking algorithm which labeled the positive samples in the first frame. Then, Babenko et al. proposed multiple instance learning (MIL) algorithm [8] which puts samples into bags. The MIL algorithm represents samples in labeled bags. The bag is labeled as positive or negative according to the contained instances. A bag is positive if at least an instance is positive, while a bag is negative when all the instances are negative [8]. The MIL algorithm learns the information from the instances in the positive and negative bags to obtain a discriminative classifier. Then, the classifier recognizes the object from background. The MIL algorithm overcomes the problem of ambiguity in object tracking.

The online MIL algorithm often fails to track an object when there is pose or illumination variations or serious occlusion. Moreover, it often suffers from computational cost. Researchers proposed some improved MIL algorithms to deal with these problems. Zhang and Song proposed a weighted multiple instance learning (WMIL) algorithm for saving the computational time [16]. The algorithm selects the most powerful weak classifiers from a classifier pool by maximizing the inner product between weak classifiers and the maximum likelihood probability, which avoids computing the bag and instance probability *M* time before a powerful classifier is selected. Xu et al. [14] selected the powerful weak classifiers by using the Fisher criterion to save the computational time. Zhou et al. [17] overcame the problem of tracking drift by computing the bag probability based on the instance importance. Wang and Zhang [18] proposed an adaptive update strategy to adjust the learning rate to deal with the problems of occlusion, pose, and illumination variations.

Therefore, a patch based multiple instance learning (P-MIL) algorithm is proposed to further improve the tracking performance and overcome the problems mentioned above. The algorithm divides an object into many blocks. Then, the multiple instance learning algorithm (MIL) is applied on each block. For each block, the positive and negative bags are composed by the instances cropped surrounding the object's position in previous frame. By training the cropped instances, weak classifiers are learned. Then, strong classifiers are generated for the blocks. The average classification score for the whole object is calculated to detect the object. Moreover, based on the average classification score and the classification scores of all the blocks, the tracking cases (occlusion, pose variations, and illumination changes) are detected and the learning rate is adjusted accordingly. Furthermore, the inner product method [16] used in WMIL algorithm is utilized to select the powerful weak classifiers for saving the computing time. Finally, we compared the P-MIL algorithm with the other MIL related algorithms in several classical videos.

The paper is organized as follows. In Section 2, we present the patch based MIL algorithm. The experiment results are illustrated in Section 3. We make a summary in Section 4.

## 2. Patch Based MIL Algorithm

**Figure 1:**
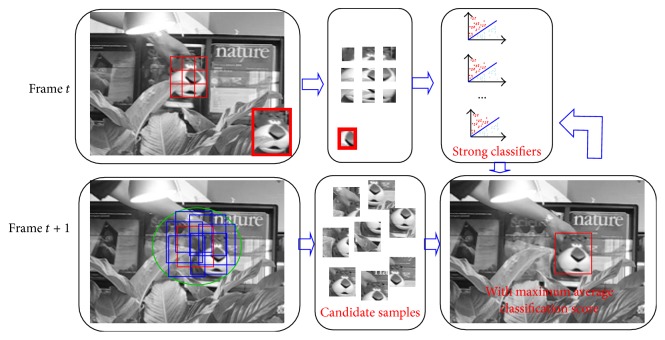
The flow chart of patch based MIL algorithm.

We proposed a patch based MIL algorithm (P-MIL) to deal with the problems mentioned above. Compared with the method representing an object in the whole, patches based method is robust to partial occlusion [19]. The P-MIL method divides an object into many blocks. Then, the online MIL algorithm is applied on each block. By training the instances in the positive and negative bags, the strong classifiers for all the blocks are obtained. In the tracking process, the obtained strong classifiers are used and the average classification scores are computed to determine the object location. Furthermore, the P-MIL algorithm detects the tracking situations including occlusion, pose changes, and illumination variations according to the average classification score and the classification scores of all the blocks. To deal with the problems mentioned above, the learning rate is adaptively tuned to update the parameters of weak classifiers. The flow chart of the proposed P-MIL algorithm is shown in Figure 1.

### 2.1. Patch Based MIL Algorithm

To realize real-time object tracking, the object is divided into 9 blocks. For block *o*_*i*_, the instances from the block and background are cropped to compose positive and negative bags, respectively. The bag is labeled “1” as it is positive, while the negative bag is assigned “0.” The positive bag is obtained by cropping instances surrounding it: *X*_*i*_^*r*_*i*_^ = {*x* : ‖*l*(*x*) − *l*_*i*_^*t*^‖ < *r*_*i*_}, *i* = 1,…, 9. *l*_*i*_^*t*^ is the position of block *o*_*i*_; *r*_*i*_ is the radius of the circle. The negative bag is obtained by cropping the instances from an annulus region around the block: *X*^*r*_*i*_,*β*_*i*_^ = {*x* : *r*_*i*_ < ‖*l*(*x*) − *l*_*i*_^*t*^‖ < *β*_*i*_}, *i* = 1,…, 9. By training the instances in the positive and negative bags, we learn the weak classifiers and obtain the classifier pool: *ϕ*_*i*_ = {*h*_*i*_^1^,…, *h*_*i*_^*k*^,…, *h*_*i*_^*N*^}, where *h*_*i*_^*k*^, *i* = 1,…, 9, *k* = 1,…, *N*, is the *k*th weak classifier for the *i*th block.

The instances in the positive and negative bags are represented by the Haar-like features [8]. It is assumed that the features of all the instances satisfy the Gaussian distribution, which means *p*(*v*_*k*_(*x*)∣*y* = 1) ~ *N*(*μ*_*k*_^1^, *σ*_*k*_^1^), *p*(*v*_*k*_(*x*)∣*y* = 0) ~ *N*(*μ*_*k*_^0^, *σ*_*k*_^0^), and *p*(*y* = 1) = *p*(*y* = 0). Then, the *k*th weak classifier with parameters (*μ*_*k*_^1^, *σ*_*k*_^1^, *μ*_*k*_^0^, *σ*_*k*_^0^) is obtained by training the *k*th feature of all the instances in the positive and negative bags:

(1) $$\begin{array}{c} h_{i}^{k}\left(\;x\;\right)=\log\left(\;\frac{p\left(y=1\;|\;v_{k}\left(x\right)\right)}{p\left(y=0\;|\;v_{k}\left(x\right)\right)}\;\right). \end{array}$$

Then, the strong classifier *H*_*i*_ is generated by selecting *K* (≪*N*) powerful weak classifiers from the weak classifier pool *ϕ*_*i*_.

(2) $$\begin{array}{c} H_{i}\left(\;x\;\right)=\overset{K}{\underset{k=1}{\sum }}h_{i}^{k}\left(\;x\;\right). \end{array}$$

**Figure 2:**
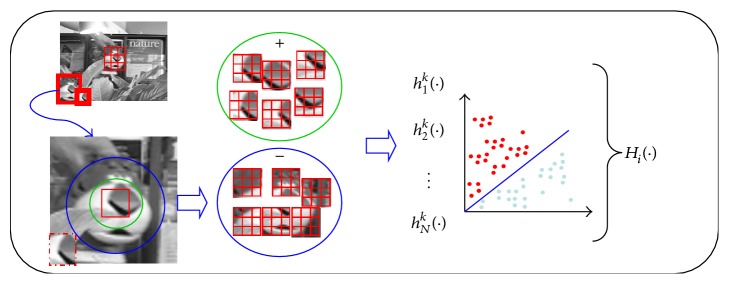
The process of applying the MIL on each block.

To save computing time, we use the inner product strategy [8] to select the powerful weak classifiers. The strategy computes the inner product between the weak classifier and log-likelihood function, which avoids computing the bag probability and instance probability *N* times before selecting a weak classifier. The process of applying the MIL on each block is shown in Figure 2.

After applying the MIL algorithm on each block, strong classifiers *H*_*i*_(*i* = 1,…, 9) are obtained and used for object tracking. In the successive frames, candidate samples *X*^*s*^ = {*x* : ‖*l*(*x*) − *l*_*t*−1_^*∗*^‖ < *s*} are extracted from the neighbor of the object location in the previous frame. For each sample, we also divide it into many blocks. Then, the classification scores of all blocks for a sample are calculated by using the corresponding strong classifiers: *S*_*i*_^*j*^, *i* = 1,…, 9, *j* = 1,…, *N*_*s*_, where *N*_*s*_ is the number of the candidate samples. The candidate sample's average classification score is obtained by averaging the classification scores of all the blocks.

(3) $$\begin{array}{c} S^{j}=\frac{1}{9}\overset{9}{\underset{i=1}{\sum }}S_{i}^{j}. \end{array}$$

The candidate sample with the maximum average classification score *S*_max_^*j*^ is considered as the tracking result.

### 2.2. The Illumination, Pose, and Occlusion Problems

The P-MIL algorithm considers the average classification score and the classification scores of all the blocks to handle the problems of illumination and pose changes and occlusion. Normally, the sample which is considered as the tracking result has the maximum average classification score and the score is greater than a given threshold. However, when the problems mentioned above happen, the maximum average classification score and the classification scores for all the blocks decrease seriously. We also see that the classification scores for all the blocks of the tracking result decrease simultaneously in case of pose and illumination variations, while the classification scores for some blocks decrease seriously in case of occlusion. Therefore, we present a method to detect the tracking situations by analyzing the classification scores for all the blocks.

We have obtained the maximum average classification score (*S*_max_^*j*^) and the classification scores for all the blocks (*S*_*i*_^*j*^) of the detected candidate sample. Then, we set two thresholds th_1_ and th_2_ (th_1_ < th_2_) as the lower threshold and higher threshold, respectively. Finally, the tracking situations are detected by analyzing the relationship between the classification score and two thresholds th_1_ and th_2_ (th_1_ < th_2_):

1. The object is successfully tracked when the maximum average classification score is greater than the higher threshold: *S*_max_^*j*^ > th_2_. In such a case, it is considered that there are not illumination changes, pose variations, and occlusion.
2. The object is occluded by the other things when the maximum average classification score is between the thresholds th_1_ and th_2_ (th_1_ < *S*_max_^*j*^ < th_2_) and the classification scores for the unoccluded blocks are greater than the threshold th_2_ (*S*_*i*_^*j*^ > th_2_  ∀*i*). In such a case, the classification scores for the occluded blocks are smaller than the threshold th_2_ (*S*_*i*_^*j*^ < th_2_  ∀*i*) (some classification scores may be smaller than the threshold  th_1_ (*S*_*i*_^*j*^ < th_1_  ∀*i*)).
3. The issues of illumination variation or pose change happen when the maximum average classification score is between the thresholds th_1_ and th_2_ (th_1_ < *S*_max_^*j*^ < th_2_) and the classification scores for all of the blocks are also between the two thresholds th_1_ and th_2_ (th_1_ < *S*_*i*_^*j*^ < th_2_).
4. The algorithm fails to track the object when the maximum classification score and the classification scores for all the blocks are smaller than the threshold th_1_  (*S*_max_^*j*^ < th_1_, *S*_*i*_^*j*^ < th_1_).
5. The algorithm fails to track the object when the maximum classification score and the classification scores for all the blocks are smaller than the threshold th_2_ for many successive frames (*S*_max_^*j*^ < th_2_, *S*_*i*_^*j*^ < th_2_).

### 2.3. Classifier Update Strategy

After tracking an object, the parameters of weak classifiers are updated to deal with the issues of illumination and pose variations and occlusion. The MIL, WMIL, and significance-MIL algorithms update their parameters with constant learning rate [5].

(4) μk1=λμk1+1−λμ1,σk1=λσk12+1−λσ12+λ1−λμk1−μ12,μk0=λμk0+1−λμ0,σk0=λσk02+1−λσ02+λ1−λμk0−μ02,

where 0 < *λ* < 1 is the learning rate. *μ*^1^ = (1/*n*)∑_*i*∣*y*_*i*_=1_*v*_*k*_(*x*_*i*_) and *μ*^0^ = (1/*n*)∑_*i*∣*y*_*i*_=0_*v*_*k*_(*x*_*i*_) are the mean values for the instances in the positive and negative bags, respectively. $\sigma^{1}=\sqrt{\left(1/n\right)\sum_{i|y_{i}=1}{\left(v_{k}\left(x_{i}\right)-\mu^{1}\right)}^{2}}$ and $\sigma^{0}=\sqrt{\left(1/n\right)\sum_{i|y_{i}=0}{\left(v_{k}\left(x_{i}\right)-\mu^{0}\right)}^{2}}$ are the variance values for the instances in the positive and negative bags, respectively.

These methods can resolve the problems mentioned above to some extent. However, it is difficult to handle the serious issues. With a large learning rate, the information of the occluding thing will be introduced into classifiers after updating. Thus, the small learning rate is helpful for the occlusion case. However, with a small learning rate, the classifier will suffer from underupdating when there is illumination or pose changes. Therefore, we present an adaptive updating algorithm to handle these problems. We have detected the tracking cases (e.g., normal tracking, illumination or pose variations, and partial occlusion) in Section 2.2. Then, different learning rates in different tracking cases are tuned as follows:

(5) $$\begin{array}{c} \lambda =\left\{\begin{array}{cc} 0.85 & th_{1}<S_{max}^{j}<th_{2},\\ & th_{1}<S_{i}^{j}<th_{2}\;\forall \;i;\\ 0.5 & S_{max}^{j}>th_{2};\\ 0.25 & th_{1}<S_{max}^{j}<th_{2},\\ & S_{i}^{j}>th_{2}\text{ for some }i. \end{array}\right. \end{array}$$

The learning rate is 0.5 in the normal case. Then, the classifier takes account of both the tracking result and the model to update its parameters. The object's appearance changes much when there is illumination or pose variations. Thus, the learning rate is set as 0.85 for updating the classifiers' parameters mainly depending on the tracking result. The classification scores of some blocks are smaller than the threshold th_2_ (even th_1_) when there is partial occlusion. The learning rate is set as 0.25 for updating the classifiers' parameters of the unoccluded blocks. For the occluded blocks, the classifiers' parameters are not updated. Therefore, the method avoids introducing information from the occluding thing. The adaptive update strategy avoids overupdating and underupdating the classifiers' parameters and resolves the issues of illumination and pose variations and occlusion.

## 3. Experimental Results

In this section, the proposed P-MIL is compared with the MIL, WMIL, and significance-MIL algorithms on several videos including “David indoor” [20], “Face occluded” [20, 21], “Tiger” [20, 21], and “Dollar” [18]. There is pose and illumination changes in the “David indoor” video. In the “Face occluded” video, the face is often occluded by a book or a hat. There are also pose variations. In the “Tiger” video, the object moves fast and is often occluded by other things. In the “Dollar” video, the object is confused by the similar features in the background. The performance of the above algorithms is evaluated in terms of tracking results, failure rate (FR), center location error, and the average computing time. All of the algorithms are implemented in the Matlab language.

### 3.1. Parameter Setting

For the online MIL boosting tracker [8], the radius for cropping instances in the positive bag and negative bag is set as *r* = 4 and *β* = 50, respectively. Thus, about 45 instances are cropped for the positive bag, while about 45 instances are for the negative bag. The MIL algorithm learns 250 weak classifiers. About 50 classifiers are selected to generate the strong classifier. For getting the final tracking result, we extract 1000 candidate samples in a circle with the radius *s* = 35 and centering at the previous object location. For the WMIL tracker [16], the instances in the positive bag are cropped in a circle with radius *r* = 4 and the instances in the negative bag are cropped in an annulus with radius *a* = 2*r* and *β* = 1.5*s*. *s* = 25 is the searching radius for extracting the candidate samples. The number of weak classifiers in classifier pool is 150. We select 15 powerful weak classifiers to generate a strong classifier for object tracking. For the significance-MIL [17], we set *r* = 4 and *β* = 50 to crop instances for the positive and negative bags, respectively. The number of the weak classifiers is 150, while that of the selected weak classifiers is 15. The learning rates for the MIL, WMIL, and significance-MIL algorithms are set to be constant: 0.85. For the P-MIL algorithm, the object is divided into 9 blocks. For each block, the number of the weak classifiers is 20, while that of the selected powerful weak classifiers is 3. To obtain the best performance, two thresholds are set as 0.8 and 0.3 as the higher and lower threshold, respectively.

### 3.2. Tracking Location

**Figure 3:**
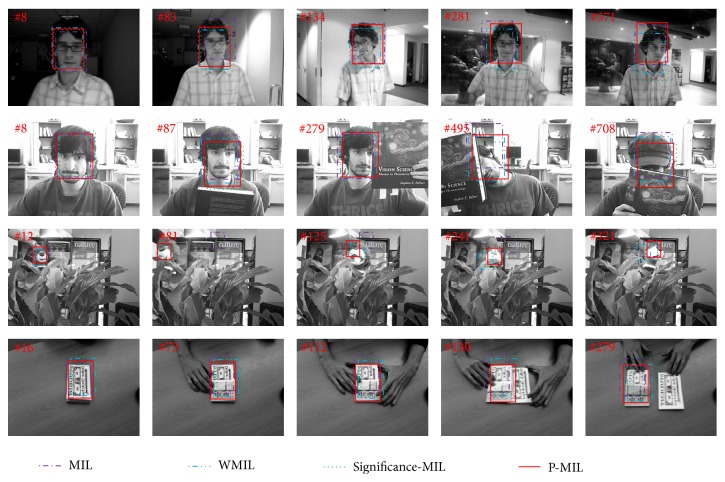
The tracking results by using the MIL, WMIL, significance-MIL, and P-MIL algorithms.

The tracking results are denoted by rectangle boxes in the images which are shown in Figure 3. The results in the first line are for the “David indoor” video. There are illumination variations (e.g., frames 8 and 83) and pose changes (e.g., frame 134). The WMIL and significance-MIL algorithms drift away in frames 281 and 371. The tracking results for the “Face occluded 2” video are shown in line 2. The “face” is frequently occluded by a book (e.g., frames 279, 495, and 708) or a hat (e.g., frames 495 and 708). Using the MIL, WMIL, and significance-MIL algorithms, the interference from background (the book and hat) is introduced into the classifier and leads to tracking drift. The “tiger” moves fast and is often occluded by leaves in the “tiger” video. The tracking results are illustrated in the third line. The MIL algorithm loses the “tiger” in frame 81 because it suffers from a heavy computation load and cannot track the fast object. The WMIL and significance-MIL methods drift away from the ground truth when occlusion occurs (in frames 241 and 321). The last line details the tracking results of the “Dollar” video. The object “Dollar” is affected by the other similar “Dollar,” which results in the fact that the MIL, WMIL, and significance-MIL algorithms drift away in frame 112. Overall, the tracking results in Figure 3 demonstrate that the P-MIL outperforms the other algorithms when there are pose and illumination variations and partial occlusion.

### 3.3. Failure Rate

We use failure rate (FR) to evaluate the performance of the MIL, WMIL, significance-MIL, and P-MIL algorithms. The tracking box with the overlap region less than 50% is considered as failure. The tracking results are shown in Table 1. The object is often affected by the issues of pose and illumination variations and partial occlusion in the tracking process. The MIL, WMIL, and significance-MIL algorithms update the classifiers with a fixed learning rate. Consequently, the classifiers are “overupdating” or “underupdating” due to the partial occlusion and illumination changes. Unlike other algorithms with a fixed learning rate, the proposed P-MIL algorithm discriminates these issues and updates the learning rate of the classifier accordingly. As a result, the P-MIL algorithm outperforms other MIL based tracking algorithms as the problems mentioned above occur. The results in Table 1 show that the P-MIL algorithm achieves the lowest failure rate.

### 3.4. The Center Location Error

**Figure 4:**
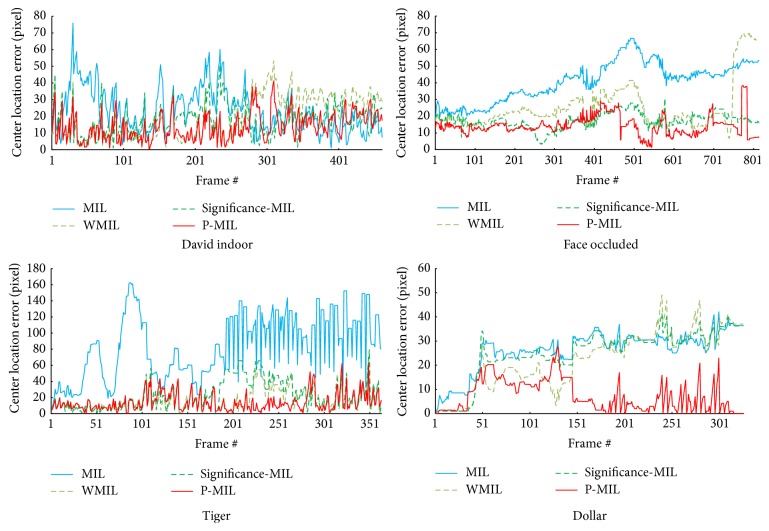
The center location error by using the MIL, WMIL, significance-MIL, and P-MIL algorithms.

This section details the center location error between central locations of the tracking results and the centers of the ground truth. The results are shown in Figure 4. The smaller the area below the center error line is, the better the tracking algorithm performs. The red lines are the center location error for the proposed MIL algorithm. The tracking results illustrate that the areas below the red lines are the smallest. The blue lines are for the MIL algorithm. The areas under the blue lines are the largest. The yellow lines are for the WMIL, while the green lines are for the significance-MIL. The results show that the P-MIL algorithm outperforms the MIL, WMIL, and significance-MIL algorithms.

### 3.5. The Average Computing Time

This section details the average computing time of the MIL, WMIL, significance-MIL, and P-MIL algorithms. The average computing time is the average processing time of a frame. The results are shown in Table 2. The lower the average computing time is, the better computational efficiency the algorithm has. The average computing time of the MIL based algorithms depends on parameters including the number of the weak classifiers in the classifier pool, the number of the selected weak classifiers, and the classifier selected strategy. The parameters in Section 3.1 illustrate that the number of the weak classifiers in the classifier pool and the number of the selected weak classifiers for the WMIL and significance-MIL algorithms are 150 and 30, which are smaller than those of the MIL algorithm. Furthermore, the WMIL and significance-MIL algorithms present efficient classifier selection strategies to avoid computing the bag probability and instance probability *M* times before selecting powerful weak classifiers, which also efficiently saves computing time. For the P-MIL algorithm, we learn about 180 weak classifiers for 9 blocks (about 20 weak classifiers are learned for a block), of which 27 weak classifiers are selected (for a block, we select 3 weak classifiers to generate a strong classifier). Moreover, we employ the weak classifier selecting strategy used in the WMIL algorithm. Therefore, the P-MIL algorithm avoids suffering from a high computational load. The results demonstrate that the P-MIL algorithm realizes real-time object tracking.

## 4. Conclusion

A patch based MIL algorithm was proposed to resolve the issues of illumination and pose variations and partial occlusion. The object was divided into 9 blocks and the MIL algorithm was applied on each block for generating 9 strong classifiers. Furthermore, the strong classifiers were used to detect the sample with the maximum average classification score as the tracking result. By analyzing the average classification score and classification scores of the sample's blocks, the illumination, pose, and occlusion problems were detected. Moreover, an adaptive learning rate update strategy was presented to avoid overupdating or underupdating. The strategy varied the learning rate based on the detected tracking situations. Thus, the updated classifiers overcome the influence from the issues of pose and illumination variations and partial occlusion. Finally, the experimental results have shown that the proposed P-MIL algorithm outperformed other related MIL algorithms in terms of computational efficiency and ability of overcoming the problems of illumination and pose variations and partial occlusion.

The object tracking algorithms are often evaluated in the classical videos which are from the Internet. However, for real videos, for example, the video from the surveillance, there is noise and the images are with low resolution. These algorithms often fail to track the object successively. Therefore, we will focus on learning a robust tracker for real videos in the future.

The MIL related algorithms cropped candidate samples around the tracking position in the previous frame. When the object moves fast, the algorithms will lose the object. To deal with the problem, we will consider motion prediction algorithm in the MIL frame.

## Figures and Tables

**Table 1:** The FR (%) by using the MIL, WMIL, significance-MIL, or P-MIL algorithm.

	MIL	WMIL	Significance-MIL	P-MIL
David indoor	14.29	0	1.421	0
Face occluded	16.05	8.21	0.798	0.24
Tiger	23.13	12.14	0.431	0.42
Dollar	0	0	0	0

**Table 2:** The average computing time (ms) by using the MIL, WMIL, significance-MIL, or P-MIL algorithm.

	MIL	WMIL	Significance-MIL	P-MIL
David indoor	1.122	0.108	1.031	0.112
Face occluded	1.601	0.104	0.123	0.097
Tiger	1.092	0.104	1.091	0.121
Dollar	1.156	0.102	0.982	0.085
